# Factors influencing the length of the incision and the operating time for total thyroidectomy

**DOI:** 10.1186/1471-2482-12-15

**Published:** 2012-07-31

**Authors:** Fabrizio Consorti, Francesca Milazzo, Mariagiovanna Notarangelo, Laura Scardella, Alfredo Antonaci

**Affiliations:** 1 Department of Surgical Sciences, University “Sapienza” of Rome, Rome, Italy

## Abstract

**Background:**

The incision used for thyroid surgery has become shorter over time, from the classical 10 cm long Kocher incision to the shortest 15 mm access achieved with Minimally Invasive Video-Assisted Thyroidectomy. This rather large interval encompasses many different possible technical choices, even if we just consider open surgery.

The aim of the study was to assess the correlation between incision length and operation duration with a set of biometric and clinical factors and establish a rationale for the decision on the length of incision in open surgery.

**Methods:**

Ninety-seven consecutive patients scheduled for total thyroidectomy were prospectively evaluated. All operations were performed by the same team and the surgeon decided the length of the incision according to his personal judgement. Patients who had previously undergone neck surgery were excluded.

**Results:**

The length of the incision was strongly correlated with gender, thyroid volume, neck circumference and clinical diagnosis and weakly correlated with the body mass index. Operation duration was only weakly correlated with gender and neck circumference. Multiple linear regression revealed that the set of factors assessed explained almost 60 % of the variance in incision length but only 20 % of the variance in operation duration. When patients were classified according to the distribution of their thyroid volume, cases within one standard deviation of the mean did not show a significant difference in terms of operation duration with incisions of various lengths.

**Conclusions:**

Although thyroid volume was a major factor in driving the decision with respect to the length of the incision, our study shows that it had only minor effect on the duration of the operation. Many more open thyroidectomies could therefore be safely performed with shorter incisions, especially in women. Duration of the operation is probably more closely linked to the inherent technical difficulty of each case.

## Background

The classical Kocher incision for thyroid surgery, which is approximately 10 cm long, has been the gold standard for more than a century. Since the introduction of Minimally Invasive (MI) surgery of the neck in the second half of the 1990s [1], several different techniques have been proposed, which have been classified as pure endoscopic techniques, video-assisted techniques and minimally invasive open surgery [2]. Recently the concept of “minimally invasive” has been questioned [3], which shows how still lively is the debate about the way to obtain satisfactory cosmetic results and limit the overall invasiveness of the procedure without enhancing the risk among patients. In the context of open surgery, different technical solutions have been proposed in the pursuit to shorten the incisions, including the use of sections of strap muscles or flapless incisions [4-6].

If we consider a 3 cm. long incision as the upper threshold of video-assisted or endoscopic techniques and 8 cm. as the lower threshold of the conventional Kocher incision, a range of many possible choices is delimited. In this context, despite some published papers [7,8], a standardised classification and guidelines to determine the appropriate extent of open access remain to be elaborated.

This study is a single-surgeon prospective survey that aimed to assess the correlation of both incision length and operation duration with a set of biometric and clinical factors and establish a rationale for the decision on the length of the incision in open surgery.

## Methods

Ninety-seven consecutive patients scheduled for a total thyroidectomy were prospectively evaluated. Patients with previous neck surgery were excluded. All operations were performed by the same medium-high volume team according to Ho [9], with a flow of approximately 100 total thyroidectomy/year. For each patient we recorded the body mass index (BMI), circumference of the neck (NC), distance between the suprasternal notch and thyroid cartilage (STD), volume of the thyroid gland (VT) as measured by ultrasound according to Ruggieri [10], length of the incision (LI), as well as the clinical and pathological diagnoses. The sample was composed by 77 female and 22 male patients, with a mean value ± s.d. for age of 54.6 ± 14.5, for BMI of 27.07 ± 5.2, for NC of 38.8 ± 4.3, for STD of 7.2 ± 1.3 and for TV of 27.7 ± 18.9. Clinical diagnoses were multinodular goitre (77), papillary carcinoma (12), hyper functioning goitre (8). In 26 cases a concurrent chronic thyroiditis was present. The duration of the operation (DO) was measured from the moment when the incision was made until the time of wound closure.

### Surgical technique

The surgeon (AA) decided the length of the incision according to his personal judgement, as determined by the patient’s clinical data. An incision was made transversally between the cricoids and the suprasternal notch. The LI was then measured, the platisma was divided and superior and inferior flaps were raised. The strap muscles were separated longitudinally and the gland was exposed. Thyroidectomy was then performed according to the conventional technique through the use of a harmonic dissector. Traction of the ipsilateral lobe outside of the wound, and lateral retraction of the lateral wound margins by retractors were used to obtain a satisfactory exposure of the surgical field, even in cases of large multinodular goitres.

### Statistical methods

All data were prospectively collected and stored in an electronic format. Comparison between means was performed by two-tailed Student’s *t*-test for unpaired samples when two variables were compared and by analysis of variance (ANOVA) for more than two variables. The strength of correlation between variables was assessed by Pearson’s *r* coefficient, while the overall predictivity of a set of factors for a dependent variable was computed by linear multiple regression. A correlation coefficient >0.50 was considered to be a sign of a strong correlation. The *r* squared (R^2^) value was assumed to be a measure of the amount of variance of the dependent variable that was explained by the independent variable or by the regression model.

## Results

**Figure 1:**
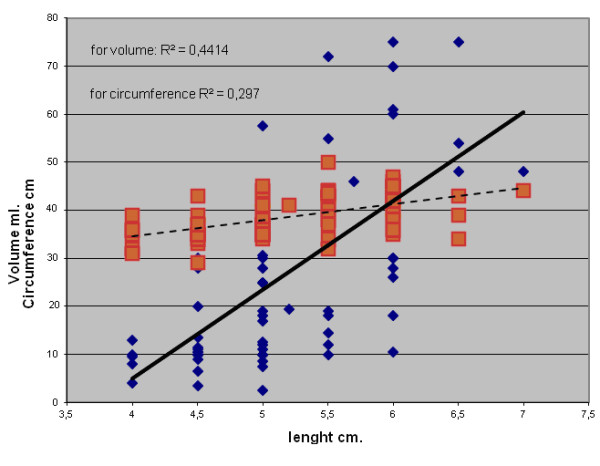
**Correlations between length of incision (LI)/thyroid volume and length of incision/neck circumference.**This figure shows the correlations between length of incision (cm.)/thyroid volume (ml.) in diamonds and bold line and length of incision (cm.)/neck circumference (cm.) in squares and dotted line. Thyroid volume explains 44 % of variance in LI, while neck circumference almost 30 %.

Length of the incision as a dependent variable was strongly correlated with thyroid volume and neck circumference and weakly correlated with BMI. The duration of the operation was only weakly correlated with neck circumference. LI and DO were also weakly related to each other. Table 1 reports the matrix of *r* coefficients, while Figure 1 shows the two strongest correlations for LI.

**Table 1:** Matrix of correlation coefficients for length of incision/duration of operation and assessed factors

	** *Age* **	** *BMI* **	** *NC* **	** *STD* **	** *TV* **	** *LI* **	** *DO* **
*Length*	0.18	0.33^a^	**0.54**^a^	−0.05	**0.66**^a^	–	0.33^a^
*Duration*	−0.23	0.08	0.33^a^	0.14	0.18	0.33^a^	–

(LI: length of incision; DO: duration of operation).

a : p < 0.05 Stronger correlations (r > 0.5) highlighted in bold.

Both LI and DO were significantly longer in men than in women and LI was also different for different diagnoses and in case with concurrent thyroiditis (Table 2).

**Table 2:** Differences in LI and DO for gender and diagnosis

	** *Length (cm)* **	** *Duration (min)* **
*M*	5.8 ± 0.56^a^	122.5 ± 29.5^a^
*F*	5.1 ± 0.64	96.0 ± 21.88
*Multinodular goiter*	5.36 ± 0.55^a^	103.0 ± 26.7^b^
*Hyperfunct*.—*Graves*	5.56 ± 0.94	105.6 ± 21.8
*Papillary carcinoma*	4.5 ± 0.55	90.0 ± 20.6
*Absence ofthyroiditis*	5.36 ± 0.7^a^	100.5 ± 25.0^b^
*Concurrent thyroiditis*	4.87 ± 0.53	102.0 ± 27.53

(mean ± st.dev.)

*t* test or ANOVA: a: p < 0.01; b: n.s.

Gender differences were observed also for NC (f: 37.57 ± 3.84, m: 43.59 ± 1.55), STD (f:7.01 ± 1.27, m: 7.91 ± 1.34) and TV (f: 25.89 ± 19.46, m: 36.69 ± 12.75) These differences were statistically significant (Student *t*-test p < 0.05).

The full model used for multiple linear regression

(1) LI=gender+BMI+NC+STD+TV+diagnosis

explained almost 60% of the variance in incision length (R^2^ = 0.59). When DO was considered as a dependent variable, only 20% of the variance was explained (R^2^ = 0.20).

To ensure that thyroid volume was similar among patients operated upon with incisions of varying lengths and a range in DO, the patients were stratified into three classes according to the distribution of their thyroid volume: cases with TV smaller than 1 standard deviation from the mean, cases within one standard deviation of the mean and cases with a TV greater than 1 standard deviation from the mean. DO was then compared to three classes of LI, defined as above according to their distribution around the mean. There was not a significant difference in DO among the classes of LI for any of the three strata of TV (Table 3).

**Table 3:** Duration of operation (min) for class of length of incision, stratified by class of thyroid volume (ml)

	** *Length < 1 st.dev (<4.5)* **	** *Length ± 1 st.dev (m: 5.2 ± 0.7)* **	** *Length > 1 st.dev. (>5.9)* **
*Volume* < *1 st*.*dev*. *(*<*10*. *5 ml)*	78.75 ± 17.9	90.0 ± 8.66	–
*Volume* ± *1 st*.*dev*. *(m: 33*.*26* ± *22*.*65)*	92.66 ± 18.21	101.66 ± 21.0	105.83 ± 23.14
*Volume* > *1 st*.*dev*. *(*>*56 ml)*	–	106.0 ± 36.8	106.87 ± 22.0

(mean ± st.dev.)

ANOVA: differences in duration of operation for each stratum of volume were not significant.

## Discussion

In Italy, thyroidectomy is the fifth most frequently performed operation in the Departments of General Surgery [11]. It is a procedure commonly performed across the country, in both large volume centres and small hospitals. MI techniques for thyroid surgery—not only MIVAT but many other approaches [12]—are proposed with increasing frequency but they require a high level of competence to minimise the length of the learning curve, which is otherwise rather long [13]. It has been stated that the "widespread application of this technique has been somewhat limited and, for practical purposes, has been confined to high-volume surgeons who have plentiful skilled assistants” [14]. No clear advantage of MI techniques in terms of medium-long term outcomes has been demonstrated [15], it is therefore reasonable to aim for standardisation and technical advancement of the conventional open technique in order to reduce the invasiveness of the procedure.

Our observational study was based on the underlying hypothesis that there is a latent tendency to over-estimate the difficulty of the operation and to create a wider incision than is strictly needed. We empirically showed that the main factors related to the length of the incision are gender, neck circumference and thyroid volume. Diagnosis and pathology had an influence, but these factors could be relevant in an indirect way, because of their influence on thyroid volume. Neither diagnosis nor the presence of thyroiditis was related to DO. These findings are consistent with the findings of Brunaud [7], who also found a correlation of LI with BMI that was stronger than the one we found. This difference could be due to a difference in the samples. Our patients tended to be over-weighted (mean BMI = 27.07), which could have counteracted the effect of this factor. BMI as a risk factor has been studied in a large database of patients [16] and was positively correlated with a longer operation time and with higher morbidity but not to a clinically significant extent. In our opinion NC is a better candidate than BMI as an element on which to base the decision regarding the incision length, but a study specifically tailored to this goal should be designed.

We found only a weak correlation among the assessed factors, including the length of the incision (*r* = 0.33) and the duration of the operation. In particular, when patients with thyroid of similar volume were operated upon with incisions of varying lengths, the time needed for the operation was not significantly different for smaller incisions compared to the longer ones.

Terris [8] proposed a classification system for MI thyroid surgery, based on two factors: size of the largest nodule and BMI. The authors then divided the continuum of possible incision lengths (from 0 to >6 cm) into four classes. These classes were defined a *priori* and then validated by retrospectively grouping a series of 359 patients. Information on DO was not available, but the results of clustering yielded mean incision lengths of 2.0, 3.3, 4.9 and 8.3 cm for the four classes, respectively. These results coincide only partially with ours, regarding the range of incisions used (4÷7 cm). In particular, there is a wide gap between the third and the fourth class. Our work provides further elements to set guidelines to assist the surgeon in the choice of a better incision for use in open surgery and that the limits of incision length in open surgery can be lowered.

A limitation of this study was that—because of the prospective design—the surgeon knew that his decision about the length of the incision was going to be recorded; this could have altered his judgement. To limit this bias, the interim results of the on-going study were not disclosed. Comparison between the figures of the first and the last set of operations during the study showed that the performance remained relatively stable regarding mean LI and DO, as if the on-going study had not altered the surgeon’s behaviour. The decision was always made according to his subjective evaluation, without any formal decision process based on empirical data. In this sense the surgeon’s decision regarding length was a consequence of his assessment of the expected technical difficulty of the operation.

## Conclusions

Our data suggest that the hypothesis of a tendency to use longer incisions than needed is likely. More open thyroidectomies could be safely performed with shorter incisions. This is especially true for women with narrow neck and with a thyroid of small volume. The duration of operation in itself is probably more closely linked to the inherent technical difficulty of each case.

## Competing interests

The authors declare that they have no competing interests.

## Authors' contributions

FC conceived the study, performed the statistical analysis and drafted the manuscript. MN participated in the design of the study and helped to draft the manuscript. LS measured thyroid volumes by ultrasound. FM participated in coordination of the study, carried out the acquisition of data and helped to draft the manuscript. AA decided the length of the incision and cooperated to revise the manuscript. All authors read and approved the final manuscript.

## Pre-publication history

The pre-publication history for this paper can be accessed here:

http://www.biomedcentral.com/1471-2482/12/15/prepub
